# Analysis of Small RNAs in *Streptococcus mutans* under Acid Stress—A New Insight for Caries Research

**DOI:** 10.3390/ijms17091529

**Published:** 2016-09-14

**Authors:** Shanshan Liu, Ye Tao, Lixia Yu, Peilin Zhuang, Qinghui Zhi, Yan Zhou, Huancai Lin

**Affiliations:** 1 Department of Preventive Dentistry, Guanghua School of Stomatology, Sun Yat-Sen University, 56 Ling Yuan Road West, Guangzhou 510055, China; liushanshancan@163.com (S.L.); taoye18@aliyun.com (Y.T.); yuu_lx@163.com (L.Y.); pelion@163.com (P.Z.); zhiqinghui@hotmail.com (Q.Z.); zhouy10.3@163.com (Y.Z.); 2 Guangdong Provincial Key Laboratory of Stomatology, Sun Yat-Sen University, Guangzhou 510055, China

**Keywords:** dental caries, gene expression, sRNAs, *Streptococcus mutans*, virulence

## Abstract

*Streptococcus mutans* (*S. mutans*) is the major clinical pathogen responsible for dental caries. Its acid tolerance has been identified as a significant virulence factor for its survival and cariogenicity in acidic conditions. Small RNAs (sRNAs) are recognized as key regulators of virulence and stress adaptation. Here, we constructed three libraries of sRNAs with small size exposed to acidic conditions for the first time, followed by verification using qRT-PCR. The levels of two sRNAs and target genes predicted to be bioinformatically related to acid tolerance were further evaluated under different acid stress conditions (pH 7.5, 6.5, 5.5, and 4.5) at three time points (0.5, 1, and 2 h). Meanwhile, bacterial growth characteristics and vitality were assessed. We obtained 1879 sRNAs with read counts of at least 100. One hundred and ten sRNAs were perfectly mapped to reported msRNAs in *S. mutans*. Ten out of 18 sRNAs were validated by qRT-PCR. The survival of bacteria declined as the acid was increased from pH 7.5 to 4.5 at each time point. The bacteria can proliferate under each pH except pH 4.5 with time. The levels of sRNAs gradually decreased from pH 7.5 to 5.5, and slightly increased in pH 4.5; however, the expression levels of target mRNAs were up-regulated in acidic conditions than in pH 7.5. These results indicate that some sRNAs are specially induced at acid stress conditions, involving acid adaptation, and provide a new insight into exploring the complex acid tolerance for *S. mutans.*

## 1. Introduction

Dental caries are one of the most common chronic infectious diseases affecting the human population [1,2,3]. According to the Third National Oral Health Survey, the prevalence of caries in five-year-old children is 66% in China [4]. Therefore, studies on the prediction and prevention of dental caries in China are essential and urgent.

*Streptococcus mutans* (*S. mutans*), a Gram-positive bacteria and one of the main clinical pathogens responsible for dental caries, can produce acidic end products through the fermentation of dietary carbohydrates [5]. Enamel demineralization, the first step in the formation of dental caries, occurs when the pH of the local micro-ecological environment is reduced to 5.5 [6]. However, *S. mutans* can survive for a long time at pH 5.5 because of its acid tolerance, which could result in the demineralization of dentine and the formation of a carious lesion [7]. Consequently, the cariogenicity of *S. mutans* is closely related to its acid tolerance. The reported virulence genes of *S. mutans* that are crucial for its acid tolerance include *htrA*, *dnaK*, *groEL*, *uvrA*, *ffh*, *brpA*, *recA*, *relA*, *glnQ*, and *glnM* [5,8,9,10,11,12,13,14,15].

In recent years, an increasing number of studies have shown that the expression of virulence genes can be regulated by small non-coding RNAs (sRNAs) at the post-transcriptional level in bacteria. sRNAs are usually smaller than 300 nucleotides (nt) and can activate or inhibit virulence gene expression via base pairing with the target mRNAs of protein-coding genes [16,17,18]. The expression levels of sRNAs are altered in response to environmental changes such as acidic pH [19,20]. To date, a large number of sRNAs have been identified in numerous bacterial species including *Staphylococcus aureus*, *Streptococcus pneumonia*, *Escherichia coli*, *Salmonella*, *Listeria monocytogenes*, *Yersinia pestis*, *Brucella melitensis*, *Acinetobacter baumannii*, *Coxiella burnetii*, and *Mycobacterium tuberculosis* [21,22,23,24,25,26,27,28,29,30,31]. However, the study of sRNAs in *S. mutans* remains in its early stages. More than 900 possible miRNA-size small RNAs (msRNAs) were recently reported, but it remains unclear whether the sRNAs with small size in *S. mutans* regulate virulence genes under acid stress conditions [32].

Therefore, in this study, we aimed to identify sRNAs with small size (18–50 nt) in *S. mutans* induced under acid stress through a deep-sequencing approach followed by qRT-PCR verification. We hypothesize that these sRNAs may play an important role in acid tolerance. To better explore this question, two of the most expressed sRNAs (*srn*884837 and *srn*133480), as verified by qRT-PCR, were used for further study. The expression levels of the two sRNAs and five corresponding target genes related to acid tolerance under different acid stress conditions at different time points were evaluated.

## 2. Results

### 2.1. Growth and pH Drop Assay Results with an Initial pH of 5.5 

**Figure 1:**
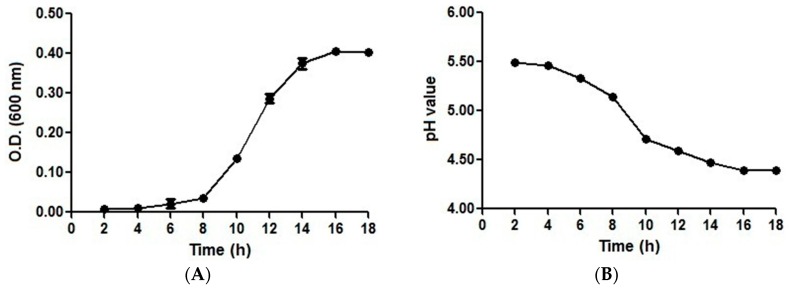
Growth curve and pH drop assay. (**A**) Growth curve of *S. mutans* cultured in brain heart infusion (BHI) broth with an initial pH of 5.5; and (**B**) the change in pH during the growth experiment. The data are presented as the means ± standard deviations from triplicate experiments.

To determine the growth characteristics and ensure that the total RNA could be extracted at the late exponential/early stationary phase, the growth curve of *S. mutans* at an initial pH of 5.5 was plotted (Figure 1A). The cells reached the late exponential/early stationary phase after 14 h of growth. This result demonstrated that *S. mutans* can continue proliferating under acid stress condition. To understand the acidic environment, the decrease in pH was then assessed. The data showed that the pH of the medium was not constant during the growth experiments and that the initial pH of 5.5 decreased to 4.4 (Figure 1B).

### 2.2. Sequence Analysis and Identification of sRNAs

**Figure 2:**
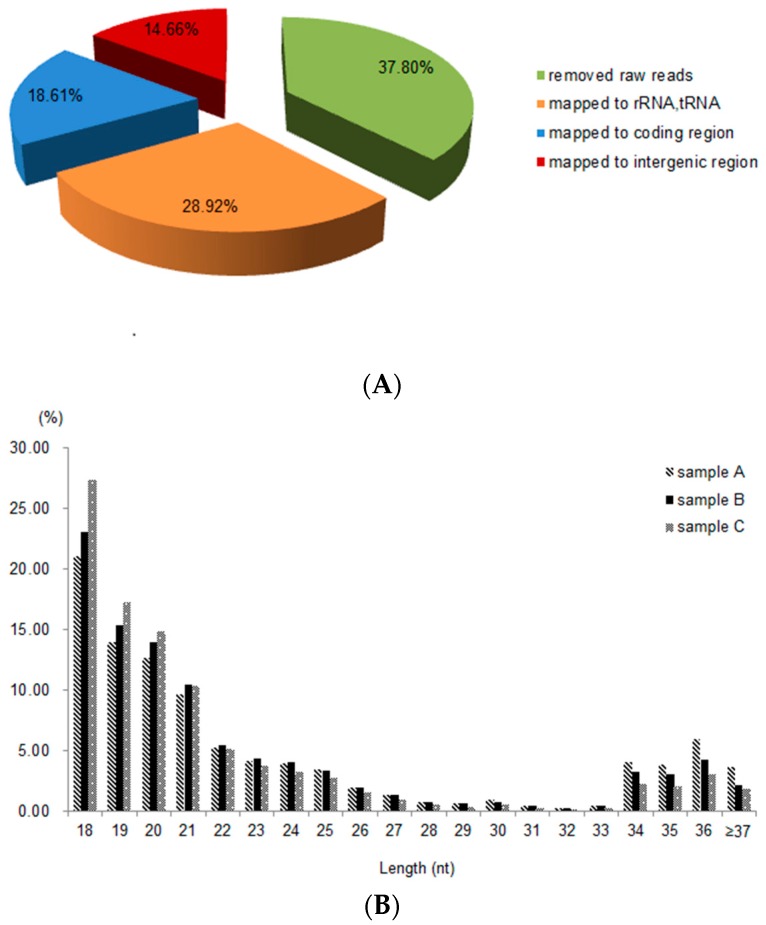
Annotation and length distribution of sequenced data. (**A**) Annotation of the constituent ratio from sequenced data; and (**B**) the length distribution of small RNA libraries in three samples. The nt lengths of sRNAs are shown on the *x*-axis; the proportion of sRNAs accounting for total unique reads are shown on the *y*-axis. sRNAs (≥37 nt in length) only account for a small proportion of the total reads and are thus shown together.

We obtained 21,784,318 average reads after sequencing. After filtering out the adapter sequences, low-quality data, and sequences shorter than 18 nt, a total of 13,549,316 average sequences among the three cDNA libraries from samples A, B, and C were obtained. Of these clean reads, 6,300,672 average sequences were mapped to ribosomal RNAs and tRNAs according to Rfam, accounting for 28.92% of the total reads. There are 4,055,147 average sequences mapped to the coding sequence, accounting for 18.61% of the total reads. Finally, a total of 3,193,407 average sequences mapped to the reference genome encoded in intergenic regions, accounting for 14.66% of the total reads, which were preserved as sRNA candidates (Figure 2A). The total length distributions of the sRNAs (mappable reads) in the three libraries are shown in Figure 2B. A total of 1879 sRNAs with at least 100 mean reads was obtained (Table S1).

For conserved analysis, only 110 preserved sRNAs were perfectly mapped to the previous study of *S. mutans* conducted by Lee et al., and no sequence was mapped to the msRNAs mentioned in the study conducted by Mao et al. [31,32] (Table S2). Some sRNAs such as *srn*236413, *srn*587174, and *srn*751903 presented higher expression levels in the current study (average counts of 220, 231, and 259), with lower expression levels in the study conducted by Lee et al.

### 2.3. Experimental Validation of Predicted sRNAs and Prediction of Target mRNA 

Eighteen putative sRNAs predicted using sequence data in Table 1 were selected for validation by qRT-PCR analysis. Among these sRNAs, 10 sRNAs were confirmed, and the high *C*_t_ value exhibited a low expression level (Table 1). To obtain preliminary information regarding the function target of these sRNAs, we predicted the target mRNAs using *RNAhybrid* for the two most highly expressed sRNAs (*srn*884837 and *srn*133480) in the current study. Surprisingly, the results suggested that the reported virulence genes responsible for the acid tolerance of *S. mutans* were putative targets of sRNAs (*glnQ*, *glnM*, *brpA*, and *relA* for *srn*884837; *ffh*, *brpA*, and *relA* for *srn*133480).

### 2.4. Bacterial Growth and Vitality Assessment under Different Acid Stress Conditions

**Figure 3:**
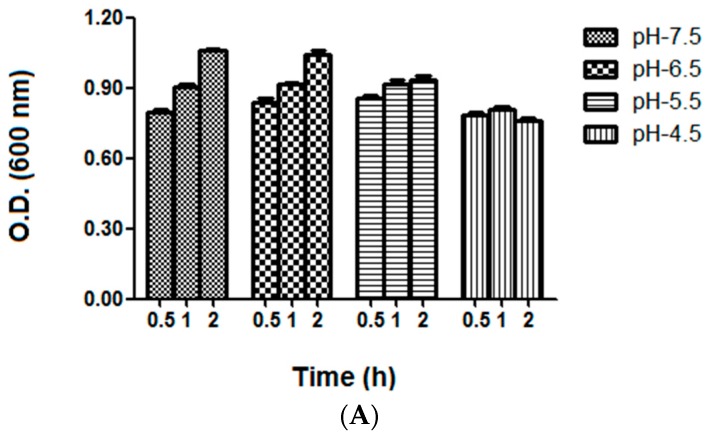

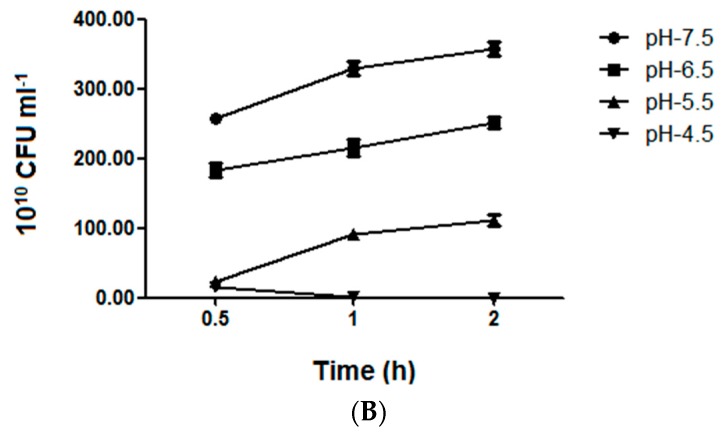
Growth and vitality analysis of *S. mutans* under different acid stress conditions. (**A**) Average absorbance (OD 600 nm) of *S. mutans* grown at pH 7.5, 6.5, 5.5, and 4.5 for 0.5, 1, and 2 h. Cells can continue to grow at a pH of 7.5 and 6.5 from 0.5 to 2 h, and pH 5.5 at the first hour (*p* < 0.05). The average absorbance value at 2 h was lower than those under pH 4.5 at 0.5 and 1 h (*p* < 0.05); and (**B**) mean number of colony forming units (colony-forming unit (CFU) × 10^10^) per mL at pH 7.5, 6.5, 5.5, and 4.5 for 0.5, 1, and 2 h. The survival bacteria at pH of 7.5, 6.5, and 5.5 increased over time and the survival bacteria at pH 4.5 decreased over time (*p* < 0.05). The survival bacteria decreased at the acid was increased from 7.5 to 4.5 (*p* < 0.001). The error bars indicate the standard deviations from triplicate experiments.

After incubation at 37 °C for 0.5, 1, or 2 h following the culture method described in the Materials and Methods section, we obtained *S. mutans* cells that can continue to grow at a pH of 7.5 and 6.5 from 0.5 to 2 h (*p* = 0.001 and *p* = 0.001). The cells were able to grow during the first hour at pH 5.5 (*p* = 0.024) and then stopped growing; however, the cell stopped growing at pH 4.5, and the OD_600_ value at 2 h was even lower than those at 0.5 and 1 h (*p* = 0.003) (Figure 3A). With regard to the assessment of *S. mutans* vitality, the changes in the number of colony-forming unit (CFU) mL^−1^ were consistent with the growth characteristics (Figure 3B). The number of surviving bacteria at pH of 7.5, 6.5, and 5.5 increased over time, whereas the number of surviving bacteria at pH 4.5 decreased over time (*p* = 0.001, *p* = 0.001, *p* = 0.000, and *p* = 0.001 for pH 7.5, 6.5, 5.5, and 4.5, respectively). At different time points, the number of surviving bacteria decreased as the acidity was increased from 7.5 to 4.5 (*p* = 0.000, *p* = 0.000, and *p* = 0.000 at 0.5 h, 1 h, and 2 h).

### 2.5. Expression Analysis of sRNAs and Target mRNAs under Different Acid Stress Conditions

**Figure 4:**
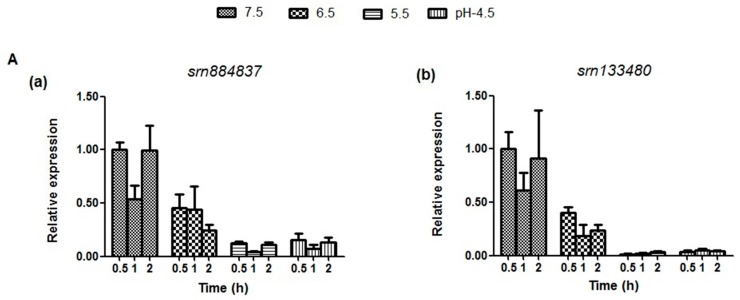

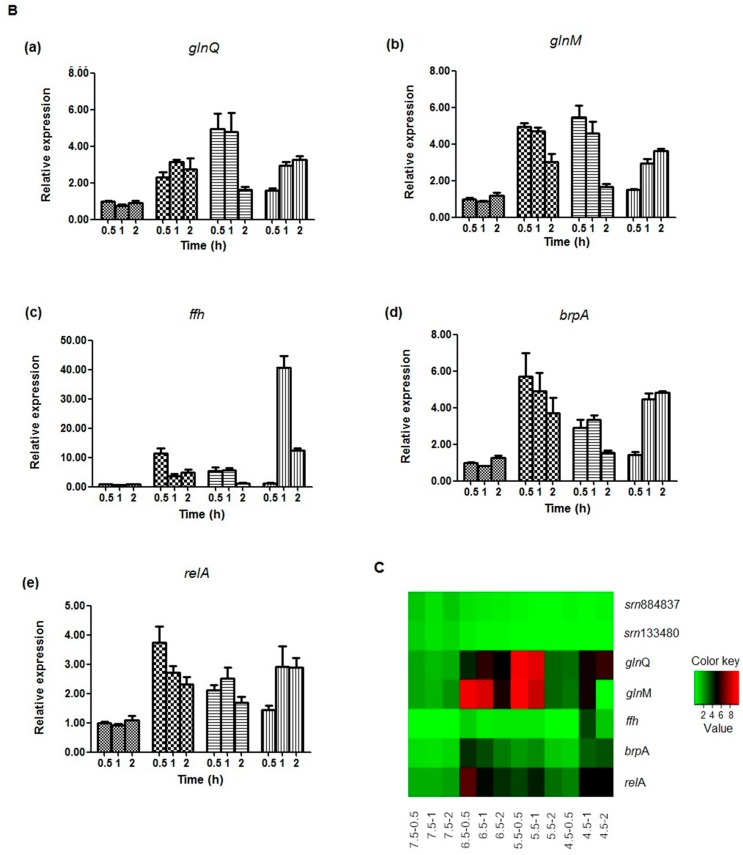
qRT-PCR analysis of sRNAs (*srn*884837 and *srn*133480) and target mRNAs (*glnQ*, *glnM*, *ffh, brpA, relA*) under different acid stress conditions. (**A**) (**a**,**b**) present the expression levels of *srn*884837 and *srn*133480 in *S. mutan,* respectively. The expression level of each sRNA after growth at pH 7.5 for 0.5 h is defined as 1.0. The error bars display the standard deviations of three biological replicates. The two sRNAs present relatively lower expression at pH of 5.5 and 4.5 compared at pH 7.5 (*p* < 0.05 for *srn*884837 and *srn*133480 at 0.5, 1, 2 h, respectively). The *p*-values for comparison of multiple means using Tukey’s honest significant difference (HSD) are detailed in Table S3; (**B**) (**a**–**e**) present the expression levels of the target genes *glnQ*, *glnM*, *ffh*, *brpA*, and *relA*, respectively. The expression level of each gene after growth at pH 7.5 for 0.5 h is defined as 1.0. The error bars display the standard deviations of three biological replicates. The target genes present relatively higher expression at pH 5.5 and 4.5 compared with pH 7.5 (*p* < 0.05 for *glnQ*, *glnM*, *ffh*, *brpA*, and *relA*, respectively). The *p*-values for comparison of multiple means are detailed in Table S3; and (**C**) a heat map showing the total relative expression patterns of the two sRNAs and five target genes at pH values from 7.5 to 4.5 at each time point. The expression level of srn133480 after growth at pH 7.5 for 0.5 h is defined as 1.0.

To preliminarily explore the biological function of sRNAs under acid stress condition, we detected the expression levels of *srn*884837 and *srn*133480, and their corresponding target genes (*glnQ*, *glnM*, *brpA*, and *relA* for *srn*884837; *ffh*, *brpA*, and *relA* for *srn*133480) related to acid tolerance under different acid stress conditions and at different time points. As shown in Figure 4A, we found that both sRNAs and their target genes can be induced during the first 0.5 h. The levels of target gene *ffh* peaked at pH 4.5, and the target genes *brpA*, *relA*, *glnQ*, and *glnM* presented higher expression levels at a pH of 6.5, 5.5, and 4.5, suggesting that *ffh* has a major function in acid tolerance, particularly at pH 4.5, and that *brpA*, *relA*, *glnQ*, and *glnM* play an extensive role in different acidic conditions (Figure 4B). Interestingly, the expression levels of *srn*884837 and *srn*133480 were lower at a pH of 5.5 and 4.5 compared with pH 7.5, whereas their target genes present relatively higher expression at pH 5.5 and 4.5 and lower expression at pH 7.5. The total relative expression levels of the two sRNAs and its target genes were shown in Figure 4C. The *p*-values for comparison of multiple means using Tukey honest significant difference (HSD) are shown in Table S3.

## 3. Discussion

Low pH value (≤5.5) can lead to demineralized tooth enamel and favor the occurrence and progression of dental caries [7]. Acid tolerance is one of the main cariogenicity factors of *S. mutans* because its acid tolerance is regarded as a particularly significant factor in its survival at low pH values [5,6,7,8]. Therefore, clarification of the survival mechanism of *S. mutans* in an acid stress environment is crucial for understanding its cariogenicity. The development of deep sequencing technology has made the identification of sRNAs feasible, and this technique has been successfully applied to the identification of sRNAs in bacterial species, including *S. mutans* [21,22,23,24,25,26,27,28,29,30,31,32,33]. Recent studies showed that sRNAs play important roles in the posttranscriptional regulation of gene expression, which can involve virulence factors [16,17,18]. However, to the best of our knowledge, few studies have focused on sRNAs with small size in *S. mutans* under acid stress conditions, particularly at different acid stress levels. Here, we describe sRNAs (18–50 nt) in *S. mutans* induced at an initial pH of 5.5. Furthermore, the vitality of bacteria and functions of two of these sRNAs under different acid conditions were evaluated.

Given that there is no common secondary structure or naming method for sRNAs, all of the reads mapped to intergenic regions of the reference genome of *S. mutans* UA159 with high sequence similarity (mismatch ≤1) from three sequencing cDNA libraries were preserved to prevent us from missing any important sRNAs. All of the sRNA candidates were named according to the starting location in the reference genome [34,35]. The results of deep sequencing and qRT-PCR suggest the existence of a family of sRNAs in *S. mutans*. Only a small proportion of sRNAs were mapped to previous studies, and the expression levels of the common sRNAs presented in this study and the previous study were not the same [32,33]. These data revealed that some sRNAs were especially induced in acid stress conditions.

Several virulence genes including *ffh*, *brpA*, *relA*, *recA*, and the rest of *S. mutans* are associated with acid tolerance [36,37,38]. It is worth noting that virulence genes (*glnQ*, *glnM*, *ffh*, *brpA*, *relA*) are a candidate target site for sRNAs according to the prediction results. *glnQ* and *glnM* are the composition of glutamate transporter operon *glnQHMP* involved in acid tolerance response (ATR) by transporting of glutamate into *S. mutans* [14]. Ffh, encoded by *ffh*, is a 54-kDa subunit homologue of the signal recognition particle of *Escherichia coli* involved in acid tolerance by altering membrane composition [6,36]. BrpA, encoded by *brpA*, is a predicted surface-associated protein with high levels of similarity to LytR of *Bacillus subtilis* and CpsX of *Streptococcus agalactiae* affects the regulation of acid tolerance [12]. The *relA* gene of *S. mutans* codes for a guanosine tetraphosphate and guanosine pentaphosphate ((p)ppGpp) synthetase/hydrolase in biofilm formation and acid tolerance [15]. These results are consistent with our hypothesis that sRNAs induced under acid stress conditions might regulate the expression level of virulence genes responsible for the acid tolerance of *S. mutans*.

The analysis of the growth characteristics and vitality indicated that cells not only survive but proliferate at pH 7.5, 6.5, and 5.5. These data are in accordance with a previous study on *S. mutans* grown at steady state in continuous culture at pH 7.0 or pH 5.0 [38]. The cells stopped growing and gradually died off at pH 4.5. The decrease in the survival capacity at pH 4.5 may be due to autolysis under acidic conditions, which has been observed for *Streptococcus pneumoniae*, another Gram-positive *Streptococcus* [39]. Target genes present relatively higher expression at pH 5.5 and 4.5 compared with their expression at pH 7.5, which induced cells’ growth and survival. Previous studies showed that genes and proteins associated with acid tolerance were up-regulated under acidic conditions, validating their importance in promoting cells’ growth and survival [36,38]. However, an opposite trend was found for sRNAs, which is consistent with the results of the survival capacity analysis. At pH 4.5, the expression levels of srn884837 and srn133480 reduced rapidly, thereby decreasing the inhibition of *glnQ*, *glnM*, *ffh*, *brpA*, and *relA*, which are essential for *S. mutans* vitality under extreme acid stress. This mechanism may be responsible for the observed phenomena. This mechanism may be responsible for the observed phenomena. These findings indicate that the expression levels of target genes are associated with sRNAs. We suspect that the expression levels of these target genes were inhibited by sRNAs. Additional experiments should be designed and performed to prove it.

Although there are some new discoveries revealed by current study it is worthwhile to point out the weakness in this exploratory study that the number of sRNAs analyzed is not enough. Further studies including more sRNAs are needed.

## 4. Materials and Methods

### 4.1. Bacterial Strains and Culture Conditions

*S. mutans* UA159 was used in this study. HCl was added to a BHI broth to adjust the pH to 5.5. *S. mutans* was incubated in BHI broth with an initial pH of 5.5 for 18 h at 37 °C under anaerobic conditions (10% H_2_, 10% CO_2_, and 80% N_2_) in anaerobic jars. The growth was monitored by measuring the absorbance at 600 nm, and the decrease in pH was assessed using a pH meter every 2 h. The experiment was performed in triplicate.

### 4.2. Total RNA Isolation

*S. mutans* UA159 was inoculated into a BHI medium at pH 5.5. Once the cells reached the late exponential/early stationary phase, total RNA was extracted and purified using the miRNeasy Mini Kit (Qiagen, Hilden, Germany), according to the manufacturer’s recommended protocol. Samples were prepared in triplicate (IDs: A, B, and C).

### 4.3. Small RNA Library Construction and Deep Sequencing

The RNAs were ligated to a 3′ RNA adapter, and then a 5′ adapter. The adapter-ligated RNAs were then subjected to reverse-transcription PCR (RT-PCR) and amplified using a low-cycle procedure. All RNA fragments that were 18–50 nt in length were isolated by PAGE according to the instructions of the TruSeq^®^ Small RNA Sample Prep kit (Illumina, San Diego, CA, USA). The library products were diluted to 10 pM for in situ cluster generation on a HiSeq2500 single-end flow cell followed by sequencing on an Illumina HiSeq™ 2500 platform (Illumina, San Diego, CA, USA).

### 4.4. Bioinformatics Analysis of Sequence Data and Identification of sRNAs

High-quality, clean data were obtained from the raw sequences after a series of data filtration steps. Rfam (http://rfam.sanger.ac.uk) was used for the removal of rRNAs and tRNAs. The remaining reads were then mapped to intergenic regions of the reference genome of *S. mutans* UA159 (http://www.ncbi.nlm.nih.gov/nuccore/AE014133) with high sequence similarity (mismatch ≤1) using the Burrows–Wheeler Alignment tool (BWA) (http://bio-bwa.sourceforge.net/) [35]. BWA, a read alignment package based on a backward search using the Burrows–Wheeler Transform, can efficiently align short sequencing reads against a large reference sequence [40]. For the analysis of known sRNAs, the sRNAs sequences were perfectly mapped to the reported msRNAs in *S. mutans* described previously [32,33].

### 4.5. Validation of sRNA Candidates and Prediction of Verified sRNA Targets

Eighteen candidates were randomly selected from those with read counts at least 100 and less than 100 respectively (Table 1). More sRNAs with read counts of at least 100 were selected because researchers usually have more interest in sRNAs of bacteria with high levels in sequencing results [32,41]. They were verified by real-time quantitative PCR (qRT-PCR). Total RNA was obtained and purified as described previously. qRT-PCR for sRNAs was performed using a mi*DETECT* A TRACK™ qRT-PCR Kit (RiboBio, Guangzhou, China) according to the manufacturer’s recommended protocol. The specific primers for sRNAs used in the qPCR assay were purchased from RiboBio. The *RNAhybrid* algorithm was previously used for the prediction of target mRNAs in bacteria [42]. It is more suitable for target prediction of short–length RNA [43,44]. The two most highly-expressed sRNAs were selected for target mRNA prediction using *RNAhybrid* with a maximum of −20 kcal/mol minimum free energy [45].

### 4.6. Effect of Different Acid Stress Conditions on the Growth, Vitality, and Gene Expression of S. mutans 

To test whether acid can affect the growth and vitality of *S. mutans* and the expression of sRNAs and their target mRNAs related to acid tolerance, we performed a previously described assay with minor modifications [20]. *S. mutans* UA159 was incubated in 36 mL of BHI broth at 37 °C until the cell reached the stationary phase of growth. The cell cultures were then divided into 12 aliquots denoted 7.5-0.5, 7.5-1, 7.5-2, 6.5-0.5, 6.5-1, 6.5-2, 5.5-0.5, 5.5-1, 5.5-2, 4.5-10.5, 4.5-1, and 4.5-2. Subsequently, these 12 samples were centrifuged and resuspended in 3 mL of fresh BHI buffer at a pH of 7.5, 6.5, 5.5, or 4.5. The buffer used in a previous study was used as a reference [46]. After incubation at 37 °C for 0.5, 1, or 2 h, 200 µL of each cell suspension was collected to measure the absorbance at 600 nm. For vitality assessment, 50 µL of each cell suspension was mixed with 450 µL of PBS. Bacterial samples (10 µL) were inoculated by spiral plating onto BHI agar plates at three dilutions (10^−6^, 10^−7^, and 10^−8^), and the total number of colony-forming units (CFUs) per volume (CFUs·mL^−1^) were quantified after anaerobic incubation for 48 h at 37 °C. The cell suspensions remaining at each time point were harvested by centrifugation and used for RNA isolation. Total RNA was extracted, and qRT-PCR for sRNAs was performed as described previously. The target genes (*glnQ*, *glnM*, *ffh*, *brpA*, and *relA*) were reverse-transcribed using the PrimeScript™ Reagent Kit (Takara, Dalian, China) according to the manufacturer’s protocol. The qPCR reaction mixture (10 µL) included 5 µL of SYBR Green Supermix (Bio-Rad, Hercules, CA, USA), 10 µM of the specific forward and reverse primers (0.2 µL for each), 1 µL of the template cDNA, and 3.6 µL of RNase-free water. The qPCR conditions included an initial denaturation at 95 °C for 20 s, followed by 40 cycles of denaturation at 95 °C for 10 s, annealing at 60 °C for 30 s, and extension at 70 °C for 1 s. The 16S rRNA levels under each condition were used for normalization. The specific forward and reverse primers for *glnQ*, *glnM*, *ffh*, *brpA*, and *relA* were described previously [14,36,37]. The primer sequences are shown in Table 2. The threshold cycle values (*C*_t_) were determined, and the data were analyzed according to the 2^−ΔΔ*C*t^ method [47].

### 4.7. Statistical Analysis

The data were analyzed using IBM SPSS 20.0 software (IBM, Armonk, NY, USA). The Shapiro–Wilk test and homogeneity of variance tests were used to assess whether the data were parametric or not. For parametric testing, one-way ANOVA followed by Tukey’s HSD test were performed for comparisons of multiple means. Values were considered statistically significant if the *p*-value was <0.05. For nonparametric testing, the Kruskal–Wallis test was first used, and one-way ANOVA was performed after data transformation when the *p*-value was less than 0.05.

## 5. Conclusions

In summary, we constructed three small non-coding RNA libraries (18–50 nt) of *S. mutans* under acid stress conditions for the first time. sRNA sequences were identified by deep sequencing combined with bioinformatics analysis. Sequences were verified using qRT-PCR. This study revealed that a series of sRNAs were uniquely induced under acid stress. The expression analysis of *srn*884837 and *srn*133480 and its target genes associated with acid tolerance suggested that some sRNAs of *S. mutans* play a critical role in acid adaptation. From the perspective of molecular biology, our novel discovery will help drive a new era of caries research.

## Figures and Tables

**Table 1:** sRNAs selected for qRT-PCR.

ID	Start Location	Length	Sequence	Strands	Mismatch	Counts (Average)	*C*_t_ (Average)
*srn*884837	1930112	29	ACGTGAATCATCGGTGCCAATACAGCATT	+	0	1143	28.47
*srn*133480	293197	27	TAAGCGATGTAAGCTGTGTGCTCTATT	+	0	2238	32.83
*srn*854592	1864362	21	AAAAAGAGCAGCTAAATCGGA	−	0	949	33.38
*srn*140177	305931	18	TTTGTTCAAGATTGTACT	+	0	170	35.67
*srn*470015	991733	19	TCTAAGACAAATTCCGTTA	−	0	114	36.27
*srn*371778	763825	18	TAACATCTGAAACTAAGG	+	0	185	36.58
*srn*821712	1793874	18	TTGACTGACTAACTATCA	+	0	35	36.90
*srn*228002	480025	20	TAGTATCTGTAGTTGCTGCA	+	0	299	37.09
*srn*638035	1357678	19	TGTCTCAGTCCTATACACA	−	0	8	37.30
*srn*219672	461702	20	GATCAATACATGTATCCTTA	−	0	48	38.38
*srn*91608	210391	18	AAGTGTCTAAGTTAGATT	−	0	139	-
*srn*174875	374257	18	GGACAGGATGTCTACTTA	−	0	101	-
*srn*342303	713515	18	GGACAGTATCTTCAATTA	−	0	177	-
*srn*430462	899609	18	AGAGTATTTAACTAGTCG	−	0	69	-
*srn*444332	929474	20	ACCAAACGATCAAACCGTGA	−	0	352	-
*srn*628738	1328165	35	ACACAGCTCTAAAACTCACCATATTAATTAATGGC	−	0	1451	-
*srn*800380	1757893	18	ATTAAGACCCCCAACAAT	−	0	436	-
*srn*821539	1793724	19	TTGTTTTAGAAACTTCTGC	+	0	44	-

**Table 2:** Primer sequences.

ID	Primer Sequences
*srn*884837	purchased from RiboBio, Guangzhou, China
*srn*133480	purchased from RiboBio, Guangzhou, China
*glnQ* (F)	GACAGGTTGTTGTTTTACTTG
*glnQ (*R)	GGTCCTTAGTTGAAGCATTGG
*glnM* (F)	GAAGTCATTCGCTCTGGTATTGAAG
*glnM* (R)	CATTGGTGGCAAGATAGTTCTGATG
*ffh* (F)	AAGGTAAGCAAGTCTCCCATTC
*ffh* (R)	TCCGTCAAATCACTGGAAAAC
*brpA* (F)	GGAGGAGCTGCATCAGGATTC
*brpA* (R)	AACTCCAGCACATCCAGCAAG
*relA* (F)	ACAAAAAGGGTATCGTCCGTACAT
*relA* (R)	AATCACGCTTGGTATTGCTAATTG
16S rRNA (F)	CTGACTTGAGTGCAGAAGGGGA
16S rRNA (R)	CGTCAGTGACAGACCAGAGAGC

## Supplementary Materials

Supplementary materials can be found at www.mdpi.com/1422-0067/17/9/1529/s1.
